# Experimental study of the temporal profile of breath alcohol concentration in a Chinese population after a light meal

**DOI:** 10.1371/journal.pone.0221237

**Published:** 2019-09-18

**Authors:** Y. C. Li, N. N. Sze, S. C. Wong, K. L. Tsui, F. L. So

**Affiliations:** 1 Department of Civil Engineering, The University of Hong Kong, Hong Kong SAR, China; 2 Department of Civil and Environmental Engineering, The Hong Kong Polytechnic University, Hung Hom, Kowloon, Hong Kong SAR, China; 3 Accident and Emergency Department, Pok Oi Hospital, Yuen Long, Hong Kong SAR, China; 4 Accident and Emergency Department, Tuen Mun Hospital, Tuen Mun, Hong Kong SAR, China; Tongii University, CHINA

## Abstract

In forensic science, the Widmark equation is widely used to deduce the blood alcohol concentration (BAC) at different time points. But the linear model specified by Widmark might be deficient in predicting the breath alcohol concentration (BrAC) at different time points, and extrapolating the peak and the corresponding time. In order to establish the temporal profile of alcohol concentration which captures the effects of non-linear nature of alcohol absorption, elimination, and peak, in particular of Chinese population after a light meal, a drinking experiment was conducted in this study. To achieve this, a double-blind drinking experiment was conducted to measure the BrAC of 52 Chinese participants after a light meal in this study. Prior to the experiment, all participants were required to abstain from food for 4 hours, more importantly, from alcohol and sedatives for 24 hours. A standard light meal was provided about 30 minutes prior to the alcohol intake in the experiment. The BrAC was measured at a 10-minute interval during the absorption phase and 30-minute interval during the elimination phase respectively. The measurements were stopped when the BrAC fell to 0.010 mg/100 ml or below, or more than 8 hours after the alcohol intake. Then, the temporal profiles of BrAC, assuming linear and non-linear relationships, were established using Full Bayesian approach. The linear component indicated the alcohol impairment in normal social function, with which a light meal is usually accompanied with drinking. On the other hand, the non-linear (gamma distribution) part replicated the absorption phase, elimination phase, and the peak of alcohol concentration. The proposed model well performed than the conventional regression model. Additionally, the confounding factors including gender, body weight, and dosage were controlled for. Results should be useful for the development of cost-effective enforcement measures that could deter against drink driving.

## Introduction

Numerous studies have demonstrated that driving under the influence (DUI) of alcohol or drugs increases the likelihood of risk-taking behavior, and thus the risk of traffic accidents and injuries fatalities [1,2,3,4,5,6,7,8]. It is therefore essential to examine the relationships between alcohol and drug doses, blood alcohol and drug concentrations, and impaired driving [9]. Many countries have attempted to combat drunk driving by imposing strict legal limits on breath alcohol concentration (BrAC) while driving. Currently, a general practice in many Western and European countries, a driver is charged with a drunk-driving offense if he or she fails a screening breath test conducted at a roadblock and a subsequent evidential breath test at a police station. Nonetheless, due to the possible time delays between non-evidential roadside screening breath test and evidential breath test at police station, which can range between 25 and 77 minutes with an average of about 40 minutes, drivers exceeding the legal limit may not be prosecuted [10]. In Hong Kong, the prescribed legal BrAC limit of 22 μg/100 ml was imposed in 2009. In December 2010, the drink-driving offense was altered to provide a three-tier penalty system in which the penalties are proportional to the BrAC level of the convicted drivers. Although the random breath test is effective in deterring drink-driving [11,12], the difference of BrAC measured between drivers who fail the screening breath test but pass the subsequent evidential breath test is considerable, because of the remarkable reduction in BrAC in the time between the administration of the two breath tests. In Hong Kong, an average time delay of about 45 minutes was recorded between February 2009 and July 2011. Further to this, between January and October 2012, 182 (24.5%) of the 744 drivers who were arrested for failing screening breath test were released and 215 (28.9%) were charged with a lighter offense than originally indicated because their alcohol concentrations dropped to a lower tier between the time of the screening breath test and evidential breath test [13]. To close this legal loophole, some policy makers are now examining the feasibility in conducting roadside evidential breath test. But considering the operational concern and the accuracy of testing machines, the roadside evidential breath test is not commonly adopted at the moment. In view, it is essential to determine a method for making a robust estimate of the BrAC of convicted drivers at the time of the roadside breath test or their conviction of a traffic offense, based on the result of the evidential breath test. In particular, the development of a temporal profile that can approximate BrAC at a previous point in time is critical.

BrAC could be measured on-site (road crash or police road block) using mobile breathalyzers. In contrast, blood alcohol concentration (BAC) is measured in the laboratory for prosecution purpose. Time lag of measurement between BrAC and BAC could be considerable. In forensic science, the Widmark equation is widely used to deduce BAC at different points in time. In this equation, the temporal profile of the BAC is dependent on the subject’s body weight and gender (the average Widmark factor, *γ*, is 0.68 for males and 0.55 for females) [14]. In addition, the mechanism of zero-order elimination rate, which assumes that the BAC decreases at a constant rate per a unit time, is commonly used to describe the elimination metabolism of the alcohol concentration. Many previous studies of alcohol elimination have adopted an average blood alcohol elimination rate, *β*, of 16 mg/100 ml/hr on an empty stomach [15,16]. Numerous experimental studies have been conducted to assess the reliability of the Widmark factors and the alcohol elimination rate in a range of subjects [16,17,18]. However, all of these studies were carried out among Caucasians in Western countries, but not for Chinese population. To review the appropriateness of the Widmark factors for a Chinese population, a drinking experiment in Hong Kong [19] was conducted using data from 149 males and 35 females. It found that the average blood alcohol elimination rate of males was 15.3 mg/100 ml/hr, and that of females was 20.0 mg/100 ml/hr on an empty stoamch. In addition, the Widmark factors for the Chinese subjects were 0.71 and 0.62 for males and females, respectively, slightly higher than for Caucasians. However, Tam’s experiment was limited by the non-randomized samples, as the participants were allowed to choose the size of the alcohol doses. No other similar drinking experiment has been conducted with a Chinese sample in recent years.

Though BAC is the most robust estimate for the alcoholic intoxication, a BrAC estimate detected with a breathalyzer, a less intrusive and more convenient instrument, is commonly adopted by the police [20]. Therefore, some studies had attempted to supplement the Widmark equation by formulating an alcohol concentration temporal profile based on the estimated elimination rate in breath alcohol [21, 22, 23]. A blood/breath alcohol ratio of 2,300:1 is usually recommended for matching the estimates of blood and breath concentration temporal profiles [24]. However, in previous studies, the subjects had empty stomachs. It is not a realistic reflection of normal situation. In particular, a meal is usually served in normal social function. Moreover, in all of these studies, the principal assumption was that the zero-order elimination rate of alcohol could be used to extrapolate the BrAC at different time points. Indeed, some researchers have argued that alcohol elimination does not always follow a linear pattern, like the one predicted by zero-order kinetics; this may be particularly the case when the BAC decreases to 20 mg/100 ml [16,25,26]. Furthermore, the linear model specified by Widmark only formulates the blood alcohol elimination phase; the absorption phase is not considered. This means that the conventional linear model might be deficient in predicting the BAC at different time points, and the time at which the maximum is attained. In order to establish the temporal profile of alcohol that characterizes the non-linear nature of alcohol absorption, elimination, and peak concentration of Chinese population in social function, a drinking experiment was conducted.

## Materials and methods

### Participants

The protocol of this study was approved by the Institutional Review Board of the University of Hong Kong, Hospital Authority Hong Kong West Cluster, and all of the participants gave informed written consent before participating. Fifty-two Chinese participants (34 males and 18 females) were recruited. The mean age and body weight of the participants was 38.2 years (ranging from 21 to 61 years) and 66.7 kg (ranging from 45.6 to 108.4 kg), respectively. The demographic characteristics of the 52 participants are summarized in Table 1. Every participant was invited to attend two or three experimental sessions, each of which was separated by 2 or more days. Prior to the experiment, the participant had to pass both a health assessment and an Alcohol Use Disorders Identification Test (AUDIT) questionnaire [27] conducted by a medical doctor. Any participants who reported having experienced alcohol or substance abuse, psychiatric disorders, or who was pregnant or breast-feeding was excluded.

**Table 1:** Distribution of the participants.

		Male	Female	Total
(a) Age Group	18–24	5	3	8
	25–34	9	7	16
	35–44	9	2	11
	45–54	7	6	13
	55 or above	4	0	4
	**Total**	**34**	**18**	**52**
(b) Weight (kg)	Below or equal to ≤ 55	1	7	8
	55 < Weight ≤ 65	11	7	18
	65 < Weight ≤ 75	12	3	15
	75 < Weight ≤ 85	8	0	8
	85 or above	2	1	3
	**Total**	**34**	**18**	**52**

### Apparatus

The Alcotest 9510 evidential breathalyzer (Drager Safety AG & Co., Germany) was used to measure BrAC. This breathalyzer quantifies the BrAC based on two separate breath samples; one is measured by infrared red (IR) sensor and the other is measured using fuel cell technology (a dual sensor technology). The maximum permissible deviation between the readings of the two sensors is 5%. In this study, the mean reading of the two sensors was recorded for subsequent analysis.

### Experiment procedures

Before the experiment, all of the participants were required to abstain from food for 4 hours and from alcohol and sedatives for 24 hours. At the beginning of the experiment, a clinical assessment of each participant’s physiological responses was conducted by a registered nurse. To account for the effect of food on alcohol metabolism, a standard light meal was provided [28]. Subsequently, the participant’s initial BrAC was measured to ensure that each participant had abstained from alcohol. About 30 minutes after the meal, the participant was asked to consume a 500 ml alcoholic drink (orange juice mixed with 40% alcohol by volume vodka) in a 15–20 minute period. The dose of alcohol was either 0, 2, 4, or 6 standard drinks. One standard drink should contain 10 g of pure alcohol and is equivalent to 100 ml of wine with 12% alcohol by volume. To reduce the risk of possible bias, the study used a double-blind procedure, in which neither the participants nor the persons administering the experiment know how much alcohol each participant had drunk. In the next step, the BrAC was measured at intervals of approximately 15 minutes (10-minute intervals during the absorption phase and 30-minute intervals during the elimination phase). The experiment ended when the BrAC fell to 0.010 mg/100 ml or below, or 8 hours after the alcohol intake.

## Statistical analysis

### Zero-order (Linear) elimination of breath alcohol

First, a baseline model of alcohol metabolites was developed based on the zero-order alcohol elimination assumption. The hourly breath alcohol elimination rate, *β*, (in mg/100 ml/hr) is a constant that is independent of the alcohol dose. The regression equation is formulated as,

$$C_{l}(t)=C_{0}-\beta t,$$ (1)

where *C*_*l*_ is the BrAC (in mg/100 ml) and *t* is the time in hours after the start of drinking. The extrapolated BrAC, *C*_*0*_ at time zero (in mg/100 ml) is a function of body weight (*W*) in kg and size of alcohol dose (*A*) in g formulated as [27],

$$\ln\;C_{0}=\alpha_{C}+\alpha_{W}\;\ln\;W+\alpha_{A}\;\ln\;A,$$ (2)

where *α*_*C*_ is the constant, and *α*_*W*_ and *α*_*A*_ are the coefficients of the relevant factors.

Substituting Eq (2) into Eq (1), we have,

$$C_{l}(t)=e^{\alpha_{C}}{∙W}^{\alpha_{W}}∙A^{\alpha_{A}}-\beta t.$$ (3)

### Non-linear elimination of breath alcohol

As alcohol metabolites are not necessarily linear in nature, non-linear regression models were developed based on a non-zero right-skewed bell-shaped assumption. Three possible function forms: (a) gamma; (b) Weibull; and (c) lognormal were considered and formulated as follows.

- Gamma function:
  $$C_{g}(t)=s∙\frac{1}{b^{a}\Gamma (a)}∙t^{a-1}∙e^{-\frac{t}{b}}.$$ (4)
- Weibull function
  $$C_{w}(t)=s∙\frac{a}{b^{a}}∙t^{a-1}∙e^{-{\left(\frac{t}{b}\right)}^{a}}.$$ (5)
- Lognormal function
  $$C_{ln}(t)=s∙\frac{1}{\sigma t∙\sqrt{2\pi }}∙e^{-\frac{{\left(\ln t-\mu \right)}^{2}}{2\sigma^{2}}}.$$ (6)

Similarly, *C*_*g*_, *C*_*w*_, and *C*_*ln*_ are the BrAC in mg/100 ml, *t* is the time in hours after the start of drinking. The parameter *s* is governed by a boundary condition specifying that the range of BrAC is directly proportional to the alcohol dose consumed by the subject as,

s=K∙A, (7)

where *K* is the constant of proportionality and *A* is the size of the alcohol dose in g.

For the gamma and Weibull functions, *a* and *b* are the shape and scale parameter, respectively, that determine the shape, skewness, and dispersion of the model; they are specified as

$$a=\gamma_{0}+\gamma_{1}∙G+\gamma_{2}∙Y+\gamma_{3}∙W+\gamma_{4}∙A,$$ (8)

$$b=\beta_{0}+\beta_{1}∙G+\beta_{2}∙Y+\beta_{3}∙W+\beta_{4}∙A,$$ (9)

where *γ*_0_ and *β*_0_ are the constants, *γ*_1_, *γ*_2_, *γ*_3_, *γ*_4_, *β*_1_, *β*_2_, *β*_3_, and *β*_4_ are the coefficients of relevant attributes, *G* represents gender (1 for female and 0 for male), and *Y* is the age of the subject.

For the lognormal function, *μ* and *σ* are the mean and standard deviation of the distribution, respectively, and can be specified as,

$$\mu =\delta_{0}+\delta_{1}∙G+\delta_{2}∙Y+\delta_{3}∙W+\delta_{4}∙A\;\mathrm{and}$$ (10)

$$\sigma =\varphi_{0}+\varphi_{1}∙G+\varphi_{2}∙Y+\varphi_{3}∙W+\varphi_{4}∙A,$$ (11)

where *δ*_0_ and *φ*_0_ are the constants, and *δ*_1_, *δ*_2_, *δ*_3_, *δ*_4_, *φ*_1_, *φ*_2_, *φ*_3_, and *φ*_4_ are the coefficients of relevant attributes.

The deviance information criterion (DIC) is used to measure the goodness-of-fit between the Bayesian models. The lower the value of DIC, the better the statistical fit of the model.

## Results

### Demographic profile

As mentioned above, every participant was asked to attend two or three experimental sessions. An evidential breathalyzer was used to record the BrAC at frequent intervals throughout each session. One hundred and nineteen experiments (83 male and 36 female) were conducted and the distributions are summarized in Table 2.

**Table 2:** Distribution of the drinking experiments.

		Gender	Alcohol dose			
			20 g	40 g	60 g	Total
(a) By Age	18–24	Male	5	5	3	**13**
	25–34		6	9	6	**21**
	35–44		5	8	6	**19**
	45–54		7	7	5	**19**
	55 or above		4	4	3	**11**
	18–24	Female	3	4	-	**7**
	25–34		6	5	-	**11**
	35–44		3	2	-	**5**
	45–54		9	4	-	**13**
	55 or above		0	0	-	**0**
		**Total**	**48**	**48**	**23**	**119**
(b) By Weight (kg)	Below or equal to ≤ 55	Male	1	1	1	**3**
	55 < Weight ≤ 65		10	11	8	**29**
	65 < Weight ≤ 75		9	11	6	**26**
	75 < Weight ≤ 85		6	8	7	**21**
	85 or above		1	2	1	**4**
	Below or equal to ≤ 55	Female	8	5	-	**13**
	55 < Weight ≤ 65		7	8	-	**15**
	65 < Weight ≤ 75		4	2	-	**6**
	75 < Weight ≤ 85		0	0	-	**0**
	85 or above		2	0	-	**2**
		**Total**	**48**	**48**	**23**	**119**

**Fig 1:**
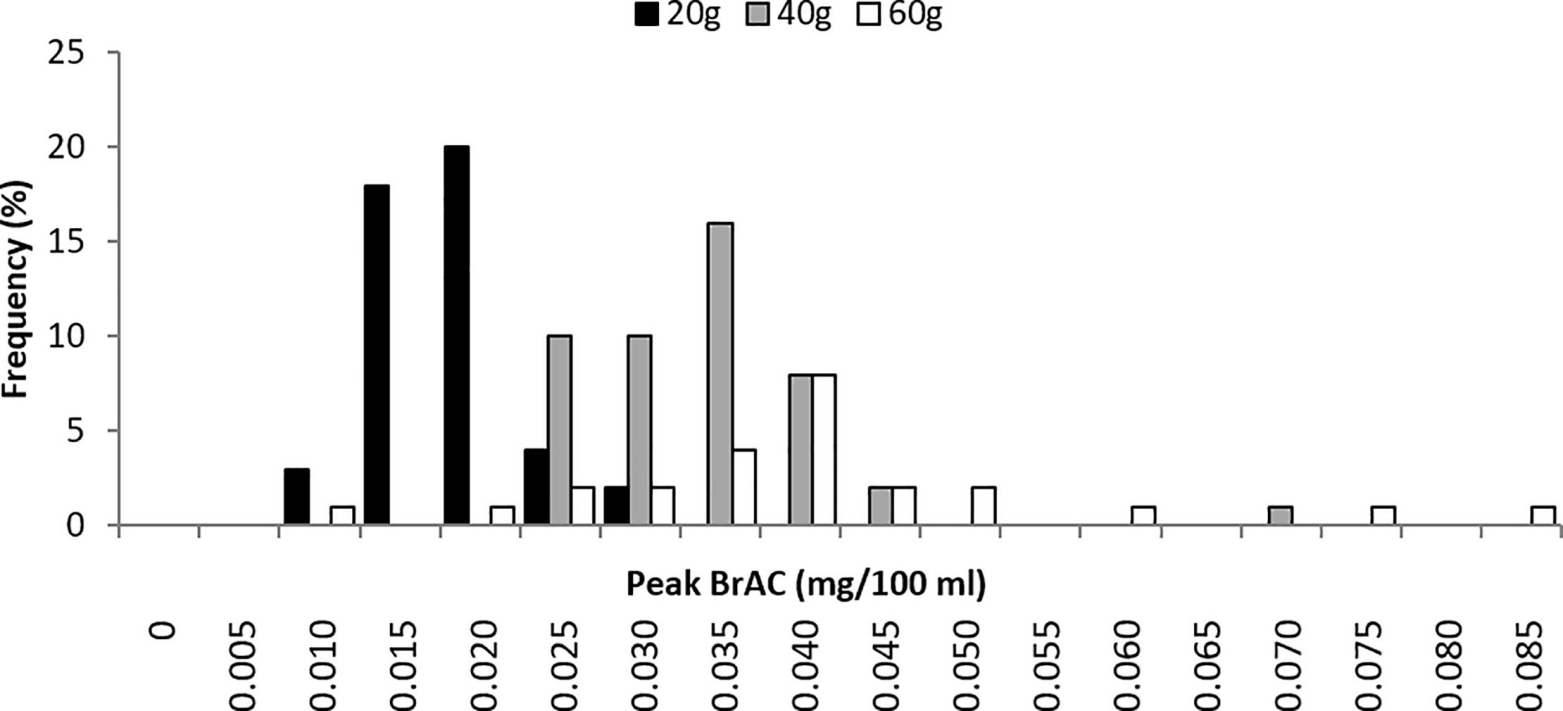
Distribution of peak BrAC for different alcohol doses.

**Fig 2:**
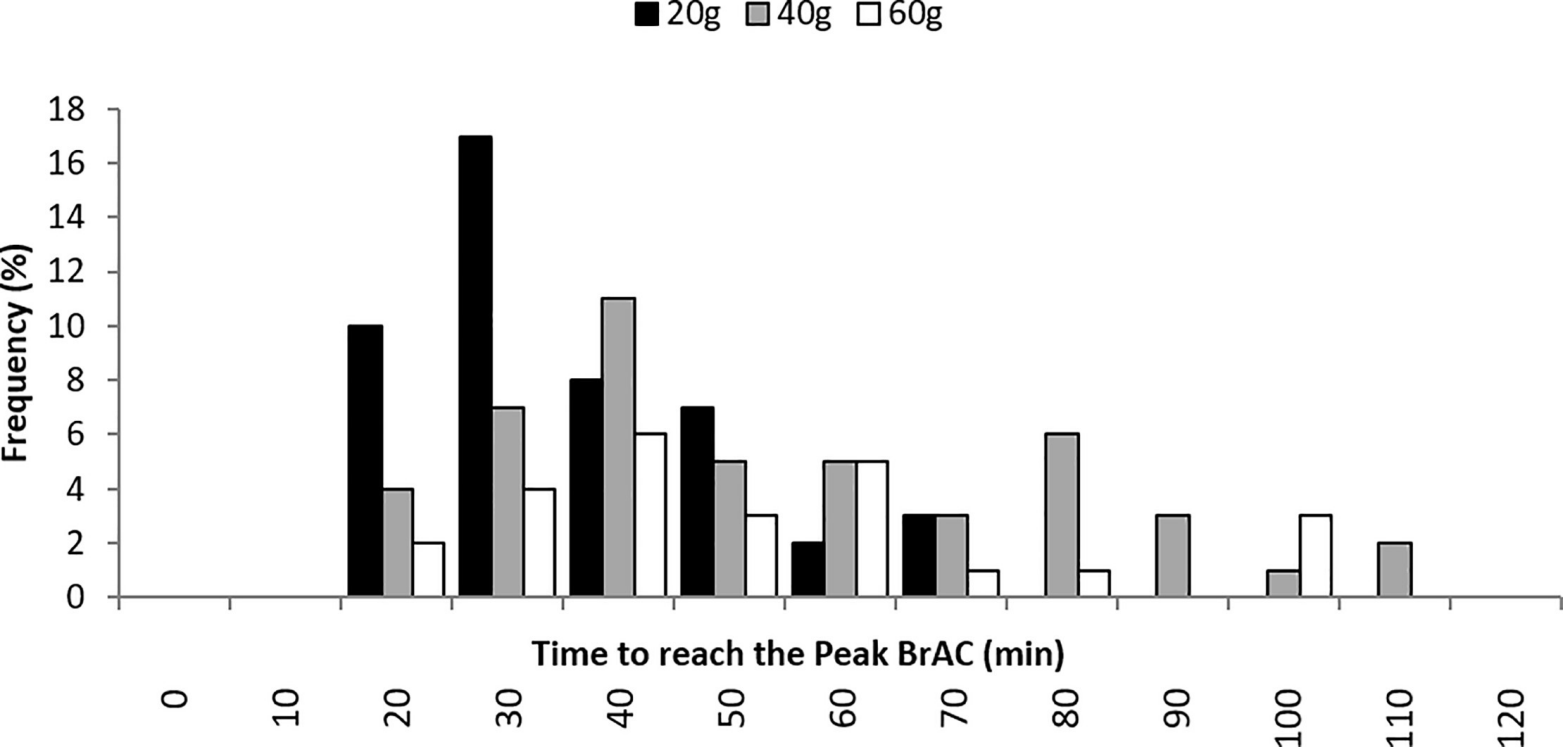
Distribution of length of time to reach peak BrAC for different alcohol dose.

The absorption phase of alcohol concentration in humans can last for 60 to 90 minutes before the peak alcohol concentration is attained [16]. Therefore, only the BrAC records at peak were used to establish the baseline model of alcohol elimination, using a linear regression approach. The distributions of peak BrACs and corresponding time, with respect to alcohol doses, are illustrated in Figs 1 and 2 respectively.

As revealed in the experiment, time between initial alcohol intake and beginning of elimination phase varied from 17 to 109 minute and the peak BrAC ranged from 0.007 to 0.081 mg/100 ml [equivalent to a BAC of 16.0 to 186.0 mg/100 ml]. As zero-order kinetics may not be applicable to extremely low BrAC, the BrAC data points with values below 0.009 mg/100 ml [equivalent to a BAC of 20.0 mg/100 ml] were excluded from the model [16]. As a result, 1,492 records were used to establish the general linear regression model.

### Zero-order (Linear) alcohol elimination

Based on the zero-order alcohol elimination assumption, a baseline model of alcohol metabolites, as specified in Eq (3), was established. Table 3 presents the coefficient estimates for the baseline model of alcohol elimination. Overall, the model fit well with the observed values and with the posterior predictive *p*-value of 0.50. The value of *C*_*0*_ increases with the alcohol dose (Parameter estimate = 0.87; 95% CI = [0.83, 0.90]), but decreases with body weight (Parameter estimate = -0.66; 95% CI = [-0.70, -0.50]), both at the 5% level of significance.

**Table 3:** Results of baseline models of zero-order alcohol elimination.

Parameter	Overall			Male			Female		
	Estimate	95% CIs		Estimate	95% CIs		Estimate	95% CIs	
		Lower	Upper		Lower	Upper		Lower	Upper
β	0.0042*	0.0041	0.0044	0.0040*	0.0040	0.0042	0.0053*	0.0050	0.0056
*α*_*C*_	2.99*	2.27	3.04	2.38*	1.45	2.82	2.06*	1.59	2.30
*α*_*W*_	-0.66*	-0.70	-0.50	-0.55*	-0.65	-0.36	-0.46*	-0.52	-0.39
*α*_*A*_	0.87*	0.83	0.90	0.90*	0.85	0.90	0.94*	0.90	0.99
Number of observation	1,492			1,105			387		
*p*-value	0.50			0.50			0.50		

* At the 5% level of significance

β is the breath alcohol elimination rate in mg/100 ml/hr

*α*_*C*_ is the constant, and *α*_*W*_ and *α*_*A*_ are the coefficients of body weight and size of alcohol dose, respectively.

Further, the breath alcohol elimination rate, *β*, was estimated to be 0.0042 mg/100 ml/hr [equivalent to a blood alcohol elimination rate of 9.7 mg/100 ml/hr], at the 5% level of significance. Not surprisingly, the breath alcohol elimination rate for females (0.0050 mg/100 ml/hr) was higher than that of males (0.0040 mg/100 ml/), which is consistent with the findings of previous studies.

### Non-linear alcohol elimination

For the non-linear alcohol elimination model, all of the 2,360 BrAC measurements, including those recorded in the absorption, peak, and elimination phases, were included in the proposed models. Table 4 presents the results of the goodness-of-fit assessment of possible non-linear alcohol elimination models with different function forms and parameter settings, i.e., it examines whether the shape and scale parameters are constant or functions of age, gender, weight, or alcohol dosage. The non-linear alcohol elimination model based on a gamma regression model where both the shape and scale parameter are linear functions of gender, age, weight, and alcohol dosage outperformed the other possible models, with a DIC value of 14,729.

**Table 4:** Goodness-of-fit assessment (DIC) of possible non-linear alcohol elimination models.

Parameter setting		Function form		
Shape parameter/ mean of lognormal function	Scale parameter/ standard deviation of lognormal function	Gamma	Weibull	Lognormal
Variable	Variable	14,729	14,825	N/A*
Variable	Constant	14,835	14,970	17,400
Constant	Variable	15,108	15,259	N/A*
Constant	Constant	15,193	15,303	N/A*

* These models could not converge

Table 5 presents the parameter estimates of the gamma models for non-linear alcohol elimination. The proposed models fit well with the observed values (the posterior predictive *p*-values are all equal to 0.50, for the overall, male, and female datasets).

**Table 5:** Results of Gamma models of non-linear alcohol elimination.

Variable	Overall			Male			Female		
	Estimate	95% CI		Estimate	95% CI		Estimate	95% CI	
		Lower	Upper		Lower	Upper		Lower	Upper
Range Parameter, *s*									
Alcohol Dose	3.46*	3.36	3.55	3.33*	3.22	3.44	3.72*	3.49	4.47
Shape Parameter, *a*									
Constant	1.28*	1.17	1.39	1.32*	1.19	1.45	0.96*	0.48	1.22
Gender (Control: Male)	0.08*	0.03	0.12	N/A			N/A		
Age	0.001	<0.001	0.002	0.001	<0.001	0.003	-0.003	-0.006	0.000
Weight	-0.004*	-0.004	-0.003	-0.004*	-0.005	-0.003	<0.001	-0.002	0.003
Alcohol Dose	0.005*	0.004	0.007	0.004*	0.003	0.006	0.016*	0.011	0.022
Scale Parameter, *b*									
Constant	1.96*	1.38	2.64	1.75*	1.06	2.51	4.24*	1.58	24.47
Gender (Control: Male)	-0.61*	-0.78	-0.45	N/A			N/A		
Age	-0.002	-0.007	0.003	<0.001	-0.007	0.007	0.003	-0.005	0.012
Weight	0.042*	0.036	0.048	0.040*	0.032	0.047	0.034*	0.024	0.045
Alcohol Dose	-0.035*	-0.043	-0.027	-0.033*	-0.041	-0.024	-0.101*	-0.594	-0.042
Number of observations	2,360			1,695			665		
*p*-value	0.50			0.50			0.50		

* Statistically significant at the 5% level

The shape and skewness of the BrAC curves were governed by the shape parameter, *a*. As shown in Table 5, a reduction in body weight (estimate = -0.004; 95% CI = [-0.004, -0.003]) and an increase in the alcohol dose (estimate = 0.005; 95% CI = [0.004, 0.007]) were associated with increases in the value of *a*, at the 5% level of significance. In addition, the value of *a* for females was higher than for males (estimate = 0.08; 95% CI = [0.03, 0.12]), at the 5% level of significance. However, there was no evidence for an association between age and the shape parameter. Based on the above estimates, the value of the shape parameter is always greater than 1 when the body weight of the subject is 75 kg or below.

Theoretically, the breath alcohol elimination rate is defined as the slope of the curve. Hence for this non-linear regression model, the breath alcohol elimination rate cab be determined by differentiating Eq (4) with respect to *t*, and have

$${C\prime }_{g}(t)=\frac{s(a-1)}{b^{a}\Gamma (a)}∙t^{a-2}e^{-\frac{t}{b}}-\frac{s}{b^{a+1}\Gamma (a)}t^{a-1}e^{-\frac{t}{b}}.$$ (12)

Again, the above equation demonstrates the breath elimination rate varied with time and is governed by the three parameters, *s*, *a* and *b*. We can further obtain the peak BrAC based on the Eq (12), such that:

When *C*′_*g*_(*t*) = 0, we have the time at which peak BrAC is reached, which is specified as,

$$t_{p}=b∙(a-1).$$ (13)

Therefore, the peak BrAC level can be estimated by

$$C_{p}=\frac{s}{b\Gamma (a)}∙{\left(a-1\right)}^{a-1}∙e^{-(a-1)}.$$ (14)

The dispersion of the BrAC value is governed by the scale parameter *b*. For instance, the variation in BrAC value decreases when the value of *b* increases, and vice versa. Also shown in Table 5, an increase in body weight (estimate = 0.042; 95% CI = [0.036, 0.048]) and a reduction in the alcohol dose (estimate = -0.035; 95% CI = [-0.043, -0.027]) were both associated with an increase in the value of *b* at the 5% level of significance. Furthermore, the value of *b* for females is lower than for males (Estimate = -0.61; 95% CI = [-0.78, -0.45]) at the 5% level of significance. Again, there was no evidence for an association between age and scale parameter. This implies that the BrAC value generally increases with the amount of alcohol, but decreases with body weight, and that the BrAC value for females is higher than for males, if all other conditions remain unchanged.

The range of BrAC is governed by the range parameter, *s*. As also shown in Table 5, the increase in the alcohol dose (estimate = 3.46; 95% CI = [3.36, 3.55]) is associated with an increase in the range of BrAC, at the 5% level of significance.

## Discussion

### Zero-order alcohol elimination

In the first part of this study, a baseline model based on the zero-order alcohol elimination assumption was developed. Consistent to previous studies, the estimated initial BrAC was positively associated with the alcohol dose, but negatively associated with body weight [14]. In this study, the estimated breath alcohol elimination rate of Chinese population (after a light meal) is 0.0042 mg/100 ml/hr [equivalent to a blood alcohol elimination rate of 9.7 mg/100 ml/hr]. This estimate is lower than that of Caucasians in Western countries (blood alcohol elimination rate of 16 mg/100 ml/hr) on an empty stomach [15,16]. In addition, the breath alcohol elimination rate of female (0.005 mg/100 ml/hr [equivalent to a blood alcohol elimination rate of 11.5 mg/100 ml/hr]) was higher than that of male (0.004 mg/100 ml/hr [equivalent to a blood alcohol elimination rate of 9.2 mg/100 ml/hr]). Since most of the previous drinking studies were conducted under empty stomach condition, this study indeed better replicate the alcohol impairment in normal social function, in which a light meal is served.

### Non-linear alcohol elimination

In the second part of this study, the prediction performance of a non-linear breath alcohol elimination model outperformed its linear counterpart. In particular, the BrAC curve was found to be of right-skewed bell-shape. To assess the effects of gender, body weight, and dosage on the maximum breath alcohol concentration and corresponding time [14,29], we took the advantage of the flexibility of a gamma regression model. In particular, the shape, range, and scale parameters are functions of human characteristics.

### Example 1: Variation in alcohol elimination by body weight

**Fig 3:**
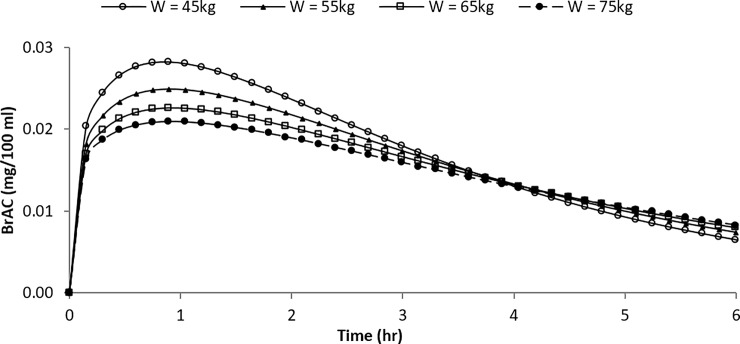
Estimated BrAC curves for different body weights (male, age 38, alcohol dose of 40 g).

Given the parameter estimates of the gamma model, curves of the predicted BrAC over time could be deduced for specified parameters defined in terms of age, weight, and alcohol dosage. The input parameters of the estimated curve for a 38 year-old male subject with a range of body weights (ranging from 45 to 85 kg), who consumes 40 g alcohol were provided in Table 6. Then, Fig 3 illustrates the curves of the predicted BrACs over time with respect to specified values of interested variables.

**Table 6:** Input parameters of the estimated BrAC curve for different body weights.

Body weight (kg)	45	55	65	75
Range parameter, *s*	138.24	138.24	138.24	138.24
Shape parameter, *a*	1.36	1.33	1.29	1.26
Scale parameter, *b*	2.37	2.78	3.20	3.61
Peak BrAC (mg/100 mg)	0.038	0.025	0.023	0.021
Time to reach peak BrAC (hr)	0.85	0.91	0.93	0.92

As shown in Fig 3, the proportional increase in the peak BrAC was less than the decrease in body weight. In addition, the peak BrAC was attained less than one hour after the alcohol intake for all of the body weights. The variation in the time that it takes to attain the peak BrAC was not sensitive to body weight. Furthermore, the differences in the predicted BrAC levels between subjects of different body weights were negligible four hours after the alcohol intake, whereas the differences in the peak BrAC level were remarkable. This indicates an urgent need for better estimates of a subject’s peak alcohol level, as evidential breath tests are usually conducted hours after an accident or a conviction in a drunk-driving case.

### Example 2: Variation in alcohol elimination by alcohol dose

**Fig 4:**
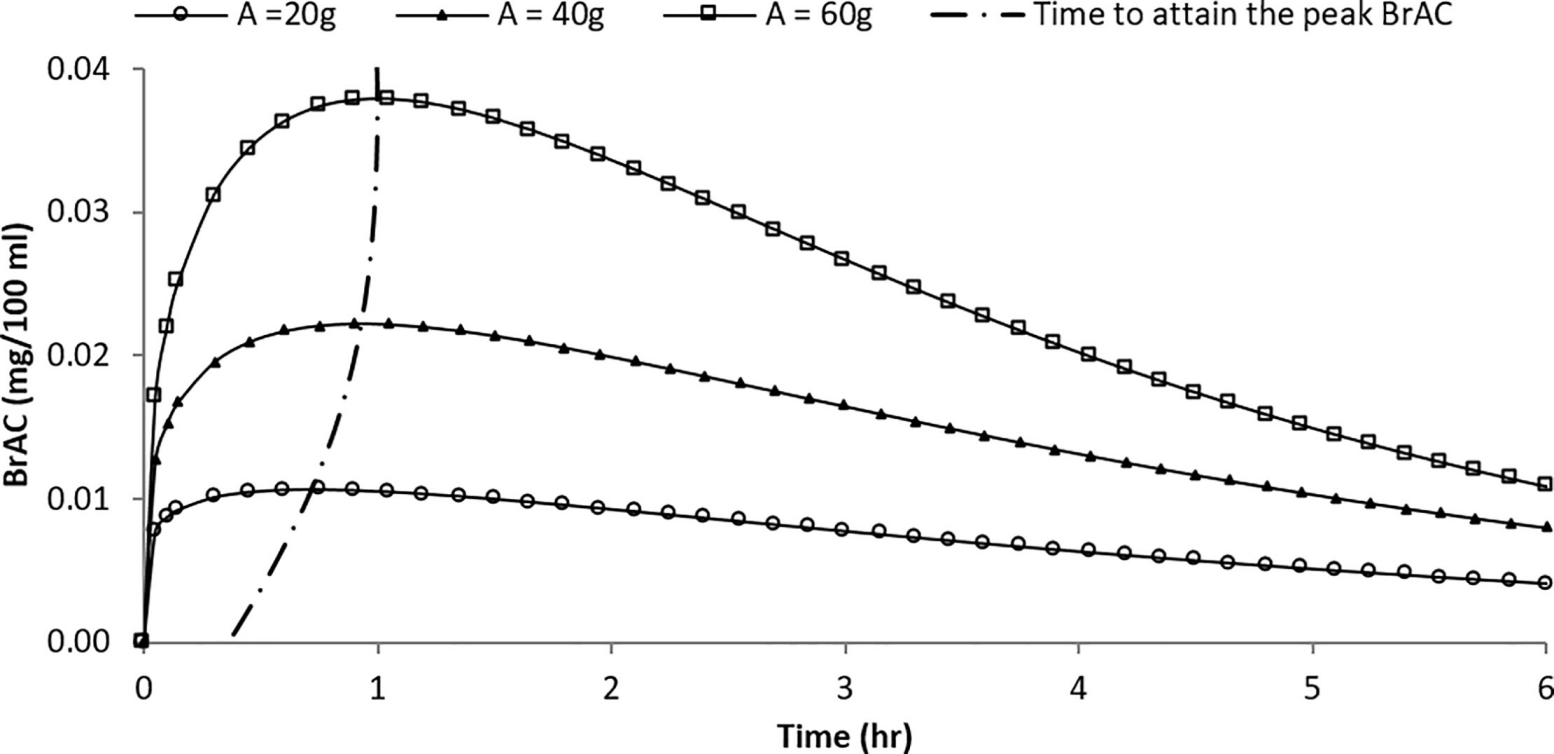
Estimated BrAC curves for different alcohol doses (male, age 38, body weight of 66.7 kg).

Similarly, the curves of the predicted BrAC over time for a 38-year-old male subject with a body weight of 66.7 kg under different alcohol doses (e.g., 20 g, 40 g, and 60 g alcohol) were deduced (Table 7 provides the input parameters of the estimated curve). As shown in Fig 4, the increase in the peak BrAC was proportionally more than the increase in the alcohol dose. Furthermore, the variation in the time that the peak BrAC was attained was remarkable across different doses. Generally, the higher the alcohol consumption, the longer it took to attain the peak BrAC. Furthermore, the differences in the BrAC levels of subjects who consumed different amounts of alcohol remained noticeable four hours or more after the initial dose. This suggests that the adverse effects of higher degrees of alcohol impairment caused by the amount of alcohol are sustained over a considerable period. Therefore, it is sensible to impose stricter punishments for seriously impaired drink drivers.

**Table 7:** Input parameters of the estimated BrAC curve for different alcohol dose.

Alcohol dose (g)	20	40	60
Range parameter, *s*	69.12	138.24	207.36
Shape parameter, *a*	1.18	1.29	1.39
Scale parameter, *b*	3.96	3.27	2.57
Peak BrAC (mg/100 mg)	0.01	0.02	0.04
Time to reach peak BrAC (hr)	0.72	0.93	1.00

### Example 3: Variation in the peak BrAC and the time until peak BrAC is attained

**Fig 5:**
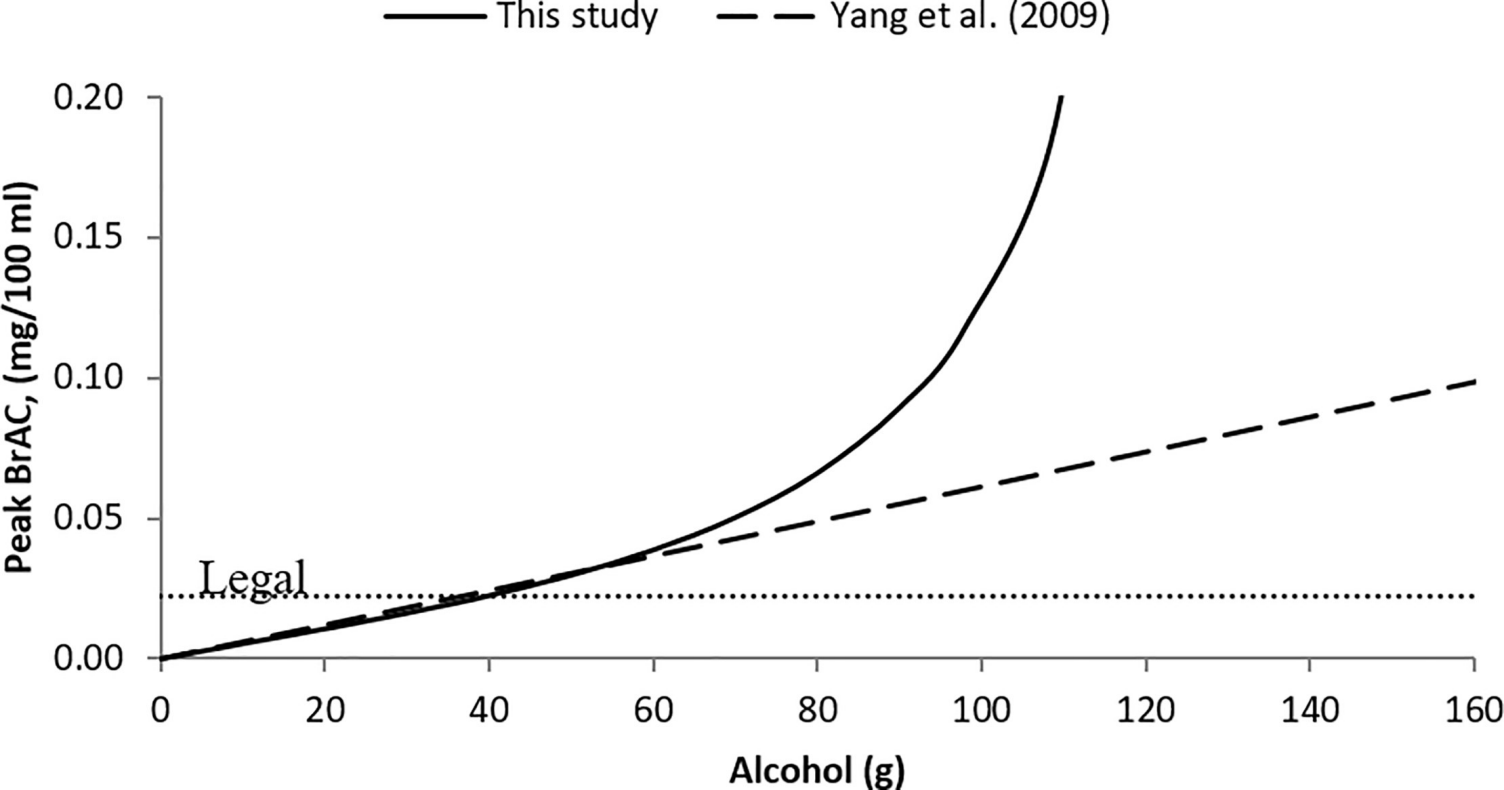
Estimated peak BrAC against amount of alcohol dose (male, age 48, body weight of 65 kg).

Based on the results using the gamma model of alcohol elimination, the peak BrAC and corresponding time can be predicted accurately using Eq (14) and Eq (13), respectively (refer to Section 4.3). Fig 5 compares the estimated peak BrAC values under different doses, according to the results of the current model and that of linear model as established in previous studies [30].

As shown in Fig 5, the difference in the peak BrAC between the current and linear models was negligible when the amount of alcohol consumption is low to moderate (ranging from 0 to 60 g). In contrast, when the amount of alcohol consumption increased beyond 60 g, the differences in the peak BrAC estimates increased substantially. Therefore, the harm caused by high alcohol consumption could be of great concern. Indeed, a drinker who consumes over 60 g of alcohol at one time is considered a high risk drinker [31]. Research by Holford stated that alcohol elimination rates in the blood are maximized when the alcohol is metabolized at its maximum rate [25]. This may be a reason why longer time is needed to attain the peak BrAC with higher alcohol consumption.

## Conclusion

In this study, a baseline model of zero-order alcohol elimination was tested. Results of parameter estimates of the baseline model were consistent to that of conventional Widmark equation. A breath alcohol elimination rate after a light meal of 0.0042 mg/100 ml/hr [equivalent to a blood alcohol elimination rate of 9.7 mg/100 ml/hr] was set out. In the second part of this study, a gamma model of non-linear breath alcohol elimination was examined. Results indicated that factors such as gender, body weight, and alcohol dose are correlated with the pattern and scale of alcohol elimination. Theoretically, non-linear model well-performed than conventional linear model in certain conditions. For example, the proposed profile is applicable to the situation where a light meal is served (not empty stomach). Considering the ethical issue, the alcohol dose was limited to 60 g only.

Moreover, this simulation study focused on actual alcohol-impaired driving performance for the general Chinese population. Results indicated that the performance of older drivers were less well in terms of the physiological responses, compared to the younger drivers. Hence, risk of alcohol-related road crashes of older drivers was higher. Same as other modern society, Hong Kong is facing the problem of ageing population (proportion of elderly population would increase to over 30% by 2035), it is worth exploring the relationship between age, driving experience, physiological response and alcohol impairment of older drivers, when more comprehensive information are available from a considerable sample of older participants in future research. This should have strong social implications to the policy strategies of driver education, licensing and enforcement.
